# CD133, Selectively Targeting the Root of Cancer

**DOI:** 10.3390/toxins8060165

**Published:** 2016-05-28

**Authors:** Jörg U. Schmohl, Daniel A. Vallera

**Affiliations:** 1 University of Minnesota Masonic Cancer Center, Section of Molecular Cancer Therapeutics, Therapeutic Radiology-Radiation Oncology, University of Minnesota, Minneapolis, MN 55423, USA; jschmohl@gmx.de; 2 Department for Hematology and Oncology, Medicine Department 2, University Hospital of Tuebingen, Tuebingen 72076, Germany

**Keywords:** cancer stem cell, CD133, relapse, BIKE, targeted therapies

## Abstract

Cancer stem cells (CSC) are capable of promoting tumor initiation and self-renewal, two important hallmarks of carcinoma formation. This population comprises a small percentage of the tumor mass and is highly resistant to chemotherapy, causing the most difficult problem in the field of cancer research, drug refractory relapse. Many CSC markers have been reported. One of the most promising and perhaps least ubiquitous is CD133, a membrane-bound pentaspan glycoprotein that is frequently expressed on CSC. There is evidence that directly targeting CD133 with biological drugs might be the most effective way to eliminate CSC. We have investigated two entirely unrelated, but highly effective approaches for selectively targeting CD133. The first involves using a special anti-CD133 single chain variable fragment (scFv) to deliver a catalytic toxin. The second utilizes this same scFv to deliver components of the immune system. In this review, we discuss the development and current status of these CD133 associated biological agents. Together, they show exceptional promise by specific and efficient CSC elimination.

## 1. Introduction

Cancer stem cells (CSC) have been a subject of interest since 1997 Dick and Bonnet identified a cell population in human myelomonocytic acute myeloid leukemia (AML). They are capable of inducing leukemic proliferation in severe combined immunodeficiency (SCID) mice after transplantation procedures [1]. In the last decade, overwhelming evidence grew confirming the existence of a cell population, which may comprise 0.1% to 20% of the tumor tissue [2], capable of promoting tumor initiation, self-propagation, and differentiation into several tumor cell entities found in carcinoma. CSCs are more chemotherapy and radiation resistant than non-CSCs, therefore explaining refractive relapse after achieved remission [3,4]. These findings led to enhanced skepticism as to whether chemotherapy alone could ultimately result in sustained high quality remissions. Among a number of CSC markers identified in several cancer entities, CD133 was identified in various carcinomas including breast, colon, prostate, liver, pancreatic, lung cancer and head and neck squamous cell carcinoma (HNSCC) [3,4]. CD133 is a membrane-bound pentaspan glycoprotein first identified in neuroepithelial stem cells in mice and later in human tissues [5]. Currently, the physiologic role of this surface receptor remains unclear. However, there is evidence of involvement in primitive cell differentiation and epidermal-mesenchymal interaction [6,7,8]. Additionally, CD133 seems to be associated with the WNT signaling pathway and thus with cell proliferation [9,10,11] and seems to be negatively associated with patient survival [12,13,14]. Analysis by Zhong *et al.* indicated correlation between CD133 overexpression, histopathological factors and poor patient outcome in hepatocellular carcinoma [15]. Several drugs have been developed to selectively target CD133 such as targeted immunotoxins capable of inducing specific drug related mortality in receptor bearing cells [16]. These drugs are antibody scFv fragments coupled to potent catalytic toxins. The scFv recognizes a marker on the cancer cell such as CD133. An scFv is the smallest variable fragment (about 20 kDA) that can be removed from an antibody that still results in antigen binding. The toxins are usually catalytic and disrupt cancer cell protein synthesis.

Early successes with immunotherapy with bispecific antibodies and T chimeric antigen receptors (CARs) have generated keen interest in immunotherapy approaches. Several studies described that modification of T-cells can enable them to express CARs and thus are able to act as powerful clinical mediators in cancer defense [17,18] for hematologic diseases, [19,20,21,22] However, modification and production of T-CARs is costly and complex. Improvement in recombinant antibody engineering made it possible to create so called immune engagers, enabling endogenous immune effector cells to target any tumor marker to which an antibody could be generated and an antibody scFv sequence could be obtained. In this context, effector cells of interest are either T-cells (adaptive immune system) or NK-cells (innate immune system). T-cells are CD3-expressing, antigen specific, and rely on peptide presentation by Major Histocompatibility Complex (MHC) I or MHC II receptors. This results in activation of killer T-cells. This mechanism is entirely circumvented by antibody dependent cell mediated cytotoxicity (ADCC). Genetically engineered bispecific immune T-cell engagers are bispecific antibodies where one antibody fragment recognizes the T-cells and the other recognizes the tumor cell resulting in the formation of an immune synaptic bridge between the effector and its target that enhances ADCC. T-cell activation is associated with cytokine toxicity that can be therapeutically problematic. Alternatively, NK-cells can also be used as immune engagers. NK-cells express CD16 (FcγRIII) and play an eminent role in tumor surveillance [23] by killing MHC class I deficient cells, following the “missing self” hypothesis [24,25].

Tumors can avoid elimination due to a variety of alterations in the human host [26,27]. As reviewed in [28], several avoidance mechanisms including resistance against cytokines, receptor downregulation, and production of immune inhibitory mediators can be circumvented using genetically engineered immune engagers. These may shift the balance towards CSC-control and precipitate anti-cancer affects. In this review, we focus on targeted toxins, NK-cell engagers targeting CD133 on CSCs and CD16^+^ immune effector cells, and some of our own laboratory work. Both constructs show promise as future alternative therapies for the prevention and treatment of chemotherapy refractory relapse.

## 2. CD133 as Cancer Stem Cell Marker

The heterogeneous cell species inside each tumor mass still remains poorly understood. Two main theories have been postulated. The older one is the “stochastic model” [29], which states that most cells inside the tumor have the same potential in dividing and renewal. Each tumor cell has the potential to reproduce the entire cancer mass. The second and leading theory is the “stem cell model” [30,31]. This dictates that a small group of stem cells are able to undergo an asymmetric cell division to either CSC or to more differentiated progenitor cells which provide more differentiated cells inside the tumor mass. Lapidot and later Dick *et al.* identified a small cell group in AML patients capable of initiating leukemia in a mouse model after injection [1,32]. These CSC showed a heterogeneous phenotype from tumor subtype to subtype. However, there are a few markers commonly expressed in CSC (Table 1), even in different types of cancer tissues [33,34]. One of these markers is CD133. CD133 mainly gained interest after experiments showing that a CD133^+^ subpopulation in a brain tumor had stem cell properties *in vivo.* After transplantation of only about 100 CD133^+^ cells into immune deficient mice, the exact same tumor was induced, whereas this was not the case for the same number of CD133^−^ cells [35,36]. The capability of a minor CD133^+^ cell fraction to induce the identical tumor after transplantation into immune deficient mice holds true for colon carcinoma [37]. Consistently, CD133 expression in high levels was shown for telomerase reverse transcriptase immortalized primary nonmalignant and malignant tumor-derived human prostate epithelial cell lines [38]. Mehra *et al.* showed enhanced CD133 receptor mRNA expression in human metastases originating from different cancer subtypes, (Table 2), [34]. CD133^+^ CSC have also been found in several other tumor types including breast, colon, prostate, liver, pancreatic, and lung cancer as well as head and neck squamous cell carcinoma (HNSCC) [3,4].

Several studies have analyzed the expression pattern of the CSC marker CD133. Due to numerous publications showing CD133 positivity in more differentiated cell types [66,70,71], questions arose if CD133 represents a good targeting choice as a CSC marker (as reviewed in [72]). The foundation of most of these findings was set in 1997 when Yin *et al.* was able to obtain a new monoclonal antibody, which bound the AC133 epitope of CD133 [73]. Explanations about different tree-dimensional refolding as a result of changes in receptor glycosylation [74] and alternative splicing of the extracellular domains of human CD133 which may affect presence as specific epitopes [75] were postulated, explaining why CD133 might not be detected in some studies analyzing differentiated cells. On the other hand several studies compared CD133^+^ with CD133^−^ cell fractions and found arguments for CD133 as a CSC marker. Tirino *et al.* used A549 cells and found a 4% expression rate in mean. After enrichment using an anti-human CD133 PE antibody expression level of CD133 was about 40% [76]. Furthermore, two distinctive groups found out that CD133^+^ colon carcinoma cells were able to initiate tumor growth, which was not visible for CD133^−^ cells [37,77]. Researchers still struggle to entirely understand the role of CD133. However this process is also supported by progress in protein engineering capable to more reliably bind CD133 related epitopes. Swaminathan, Ohlfest, and coworkers fundamentally improved the opportunities of CSC detection and developed a novel anti-human CD133 monoclonal antibody (clone 7), able to bind an unmodified CD133 extracellular domain [78]. This led to an improvement of reliability and specificity in CD133 binding and formed the backbone of new cancer targeting antibody constructs.

New therapeutic anti-CD133 antibodies are useful only if their ability to bind CSC is indisputable. Fortunately, this can be readily tested using the antibody to sort CD133^+^ cells and then injecting them as flank tumor xenografts. Enriched populations are expected to grow fast and larger while the diminished fractions grow more weakly and slowly. Waldron *et al.* [79] performed such an animal experiment with CD133^+^ cells selected from UMSCC-11B Head and Neck cancer tumor cells enriched using magnetic bead selection. CD133^+^ CSC were then inoculated in a tumor flank model and compared with enriched CD133^+^ cells pretreated with dCD133KEL (a targeted toxin which used a Clone 7 related binding site). Results showed a significantly reduced tumor growth rate in the group where CSC were depleted [79], implying efficacy in binding real CD133^+^ cells with tumor initiating CSC properties.

## 3. CD133 Expression on Normal Body Cells

Besides expression on CSC, CD133 is known to be expressed on normal hematopoietic, neuronal and endothelial progenitors [73,80,81]. Thus, targeting of CD133^+^ on normal body cells might bear the risk of potential side effects. In the bone marrow, CD133 is expressed on cells of the hematopoietic system. Since our group developed a highly specific and potent genetically engineered targeted toxin known to inhibit the growth of tumor initiating cells in cancer xenograft models, we were able to determine its effects on normal enriched human progenitor cells measured in various progenitor assays including long-term culture and colony-forming assays. Cord blood was used as a source of normal stem cells. In all of these assays, minimal effects were noted indicating that normal progenitors were not affected [79]. Several explanations are possible. 1) Perhaps CD133 is expressed in a far lower copy number in normal stem cells than in transformed CSC. CSC may express CD133 at a higher level than the normal progenitors cells, which indeed has been shown in colorectal, pancreatic, gastric, and hepatocellular carcinomas [82] thus leading to more toxicity in target cells than in physiologic progenitors. 2) Perhaps plasticity of the human hematopoietic system might ensure the absence of drug associated toxicity by selecting a normal stem cell population with a CD133^−^ phenotype still capable of differentiating into multiple hematopoietic cell types [83]. Surronen *et al.* revealed that CD133^+^ cells have plasticity meaning that CD133^+^ cells can be generated from a population of CD133^−^ cells [84]. 3) CD133^+^ cells might be generated from myeloid precursors or monocytes that act as pluripotent stem cells [85]. 4) Lack of toxicity may be based on de-differentiation which was described not only in mammalian, myeloblast and pancreatic cells [86,87], but also in late endothelial progenitors before their differentiation to mature endothelial cells [88,89]. Upon loss of the capability to express CD133, these cells might still have the potential to again express CD133.

CD133 is expressed on glioma cells in the central nervous system [35,90]. These cells were capable of extensive self-renewal, differentiation and marker expression of the astrocytic, oligodendroglial and neuronal lineage [36]. In a healthy central nervous system, CD133^+^ progenitor cells have not been identified, however, CD133-enriched membrane particles in the neural tube fluid have been detected [91]. Due to the brain-blood barrier, systemic application would probably not affect the central nervous system, because neither monospecific antibody conjugated immunotoxins nor bispecific antibodies should be able to pass due to enlarged molecular size [92,93,94]. Despite the lack of clinical data, an *in vivo* mouse model demonstrated that mice exposed to a CD133 targeted immunotoxin showed no neurotoxicity, despite continuous injections over several weeks [63]. This might lead us to conclude that CD133 target related neurotoxicity in humans might not be expected upon systemic drug treatment.

Gehling *et al.* identified CD133^+^ progenitor cells in human peripheral blood capable of differentiating into hematopoietic lineages but also into endothelial cells [95]. They showed that CD133 receptor directly interacts with the angiogenetic vascular endothelial growth factor which might be relevant for physiologic neovascularization but also for angiogenesis in cancer patients. *In vivo* angiogenesis could be inhibited by downregulation of CD133 using lentiviral short hairpin RNA sequences to silence prominin-1 gene capillary formation [96]. A study that histologically analyzed lung tissue from lung cancer patients stated that CD133^+^ endothelial progenitor cells contributed to neovascularization and tumor growth vascular damage was not reported after using CD133 targeted constructs but data implicate the potential of capillary complications after administration.

## 4. CD133 Directed Targeted Therapies, Potential and Delivery

Anti-CD133^+^ scFv conjugated targeted toxins and bispecific antibodies have to be delivered to the respective targets and therefore have to be administrated intravenously. However, vascularization of solid tumors is inherently nonhomogeneous and partly associated with hypoperfusion. The presence of large molecules such as collagen from the extravascular system, longer vessel length and reduced perfusion can limit focused delivery of the targeting drug to the point of interest [97]. Drug delivery is dependent on diffusion capacity from vessel wall to the target cell which is influenced by tumor extracellular matrix composition which has also been shown to be of heterogeneous makeup [97]. As a consequence, systemic treatment of solid tumors might prove difficult. To address this problem, especially for targeted toxins, a combination therapy of immunotoxin plus chemotherapy might improve the clinical outcome combining advantages in drug penetration of chemotherapies with a selective effect on CD133^+^ cancer stem cells. The hypothesis would be that the chemotherapy would diminish the tumor bulk of rapidly dividing cells while the CSC-targeted therapy would eliminate the subpopulation of cells at the root of chemo-resistance. To date, combination therapy with a CD133^+^ specific drug and regular systemic chemotherapy has not been described in the literature and still needs to be investigated in a clinical setting. However, one drawback could be that combined therapy may not only lead to an improved therapeutic outcome but also to enhanced drug related toxicity [98].

Hematologic cancer, or liquid tumors as they are called, provides an excellent target, avoiding problems with tumor perfusion. For example, Blinatumomab, a bispecific T-cell engager (BITE) against CD19 (a B-cell receptor) showed very good results in treatment of acute lymphoblastic leukemia [99,100] and additionally in minimal residual disease (MRD) situations [101].

## 5. Potential CD133 Targeting Immunotherapies

### 5.1. Immunotoxins

#### 5.1.1. ^C178A^BC-CD133MAb

**Figure 1:**
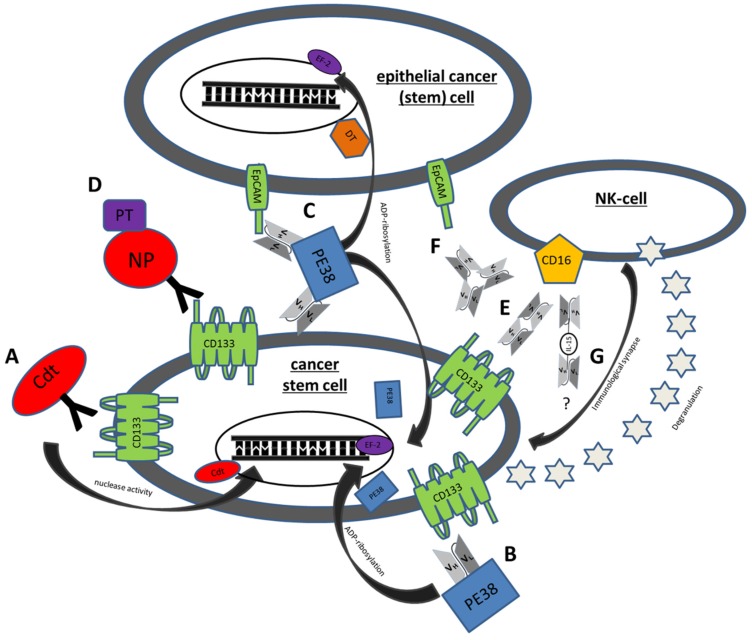
Mechanisms of action of some genetically engineered CSC Targeting Drugs. (**A**) ^C178A^BC-CD133MAb is a monospecific anti-CD133 antibody that is conjugated to cytolethal distending toxin (Cdt). This toxin has nuclease activity and induces DNA damage in host cells. **(B**) Deimmunized CD133KDEL (dCD133KDEL) consists of an anti-CD133 scFv on the same single chain molecule as truncated deimmunized pseudomonas exotoxin A (PE38). It induces ADP-ribosylation of elongation factor 2 (EF-2) leading to irreversible inhibition of protein synthesis. (**C**) Deimmunized EpCAMCD133KDEL (dEpCAMCD133KDEL) consists of two ligands, anti-CD133 scFv and anti-EpCAM scFv with the same deimmunized PE toxin. (**D**) NP133 is a nanoparticle [NP] linked to an anti-CD133 antibody and loaded with paclitaxel [PT], a microtubule-stabilizing anticancer drug. (**E**) CD16 × 133 is a bispecific scFv to target CD133 and thereby forming an immune synapse with anti-CD16 on NK-cells resulting in induction of ADCC. (**F**) 133EpCAM16 is a trispecific construct, targeting CD133, EpCAM, and CD16. (**G**) 161533 TriKE consists of an anti-CD33 and anti-CD16 scFv and a modified IL-15 linker but might also be modifiable to a 1615133 TriKE. It induces NK expansion and ADCC.

^C178A^BC-CD133MAb is a monospecific anti-CD133 antibody, conjugated to cytolethal distending toxin (Cdt) derived from Aggregatibacter actinomycetemcomitans. Cdt has nuclease activity and leads to DNA damage in host cells with consecutive growth arrest and subsequent cell death as shown in mammalian cell lines. *In vitro* studies of ^C178A^BC-CD133MAb and CD133^+^ cells derived from HNSCC patient samples showed specific and dose dependent inhibition of proliferation [64]. However, the drug never achieved Phase 1 status (Table 3, Figure 1A).

#### 5.1.2. dCD133KDEL

Our group developed a deimmunized targeted toxin called CD133KDEL, which was synthesized using an anti-CD133 scFv reactive against the extracellular domain of CD133. This scFv (clone 7) was species cross reactive [108] and had the ability to bind all CD133 isoforms [78]. The scFv was cloned onto the same molecule containing a truncated, deimmunized form of pseudomonas exotoxin A (PE38) [79] to prevent the generation of activity blocking neutralizing antibodies. PE38 is a biologic drug that is extremely potent in anticancer activity when modified and used as a targeted toxin, (Figure 1B) [109]. Upon internalization, cell killing is achieved through catalyzing ADP-ribosylation of elongation factor 2 (EF-2) leading to irreversible inhibition of protein synthesis and cell death [110]. Through addition of a Lys-Asp-Glu-Leu (KDEL) *C*-terminal sequence, activity is increased by enhancing drug presence in the endoplasmatic reticulum (ER) [111]. An advantage of ligand-directed toxins is specific ligand-receptor binding. After specific binding to the respective target structure, the drug is internalized resulting in maximal anticancer activity while limiting collateral killing of normal non-target cells. Efficacy of CD133KDEL was demonstrated using three different xenograft models by our group, despite low levels of CD133^+^ CSC. These included a head and neck cancer model where the drug was injected intraperitoneally (IP) [79], an ovarian cancer model, where both tumor and drug were injected IP [65], and a triple negative breast cancer model where the treatment was systemic [112]. Efficacy could only be sustained with weekly treatments (Table 3). Taken together, these findings show that targeting CD133^+^ can be a powerful approach in eliminating CSC at the root of carcinoma relapse.

#### 5.1.3. dEpCAMCD133KDEL

Since CD133 reacts with only a small, albeit important, population of cancer cells, there may be advantages of using anti-CD133 scFv (clone 7) in combination with scFvs recognizing other tumor markers. There are important advantages to targeting 2 cancer markers simultaneously: 1) Immune escape mechanisms such as shedding of tumor associated antigens may complicate therapy; 2) the tumor mass consists of a heterogeneous texture of different cell subtypes with different importance for renewal and progression. Multiple binding sites can attack these sites simultaneously and create synergistic effects; 3) targeting two binding sites on one target can lead to an improved affinity. Thus, we synthesized dEpCAMCD133KDEL a single chain targeted toxin consisting of an anti-CD133 scFv linked to an anti-EpCAM scFv and the deimmunized, truncated form of PE38, (Figure 1C). EpCAM is a well-established CSC target which has diverse roles in cancer cells, such as cell signaling, proliferation, differentiation, and migration [113,114]. Recent data linked EpCAM to WNT/β-catenin signaling, which represents a key pathway in both cancer stem cells and normal adult stem cells and plays an important role in self-renewal and differentiation. Furthermore, high levels of EpCAM expression correlate with increased tumorigenesis in various carcinomas [115,116]. In efficacy studies of this bispecific toxin, our group tested dEpCAMCD133KDEL in nude mice with UMSCC-11B head and neck tumors tagged with luciferase. Tumors in groups treated with dEpCAMCD133KDEL showed regression with complete remission (CR). Control groups showed tumor progression. Furthermore, efficacy of dEpCAMCD133KDEL was shown in breast and colon carcinoma cell lines *in vitro*. To compare efficacy of a monospecific to a bispecific drug another group of mice was treated with anti-CD133-targeted toxin. Data showed tumor response was greater in the bispecific treatment [103] (Table 3). Taken together, multispecific targeting with inclusion of CD133 as a binding site shows promise in eliminating carcinoma and inhibiting tumor progression/renewal and drug refractory relapse.

#### 5.1.4. Anti-CD133 Conjugated Nanoparticles

Swaminathan *et al.* [104] focused on nano-particles conjugated with an anti-CD133 antibody (clone 7), loaded with paclitaxel, a microtubule-stabilizing anticancer drug, (Figure 1D). *In vitro* the drug showed efficacy in targeting Caco-2 cell lines and elimination of mammospheres, which are routinely used as a quantitative model of CSC [117]. For *in vivo* studies, BALB/c nu/nu mice were used in an othotopic breast cancer model (MDA-MB-231 transfected with luciferase). Results showed better tumor decrease after treatment with nanoparticles compared to controls. Furthermore, relapse occurred less frequently in the group treated with the construct [104].

### 5.2. BiTES

#### 5.2.1. MS133

Harnessing T-cells for immunotherapy against malignancies has emerged as effective since bispecific T-cell engagers (BITES) are capable of supporting a link between tumor associated antigen bearing tumor cells and CD3^+^ T-cell effectors [118]. Different BiTES are already established in the clinical routine. Blinatumomab, a bispecific scFv antibody binding CD19 and CD3, showed success in treatment of relapse or refractory B-precursor acute lymphoblastic leukemia [99,100,101,119]. Catumaxomab, a bispecific scFv antibody targeting EpCAM on tumor cells and CD3 on T-cells, showed efficacy in the clinical treatment of carcinoma related malignant ascites [120]. Zhao *et al.* [106] used this mode of action to target CD133^+^ carcinoma. They developed an asymmetric bispecific antibody (MS133) consisting of a binding site against CD133 and against CD3 and showed tumor cell elimination in CD133^+^ colorectal carcinoma cell line (HCT116) *in vitro*. Furthermore, repression of tumor initiation in a NOD/SCID tumor model is observed when activated T cells were administrated [106].

#### 5.2.2. Anti-CD3/CD133 Bispecific Antibody

Huang *et al.* [107] engineered a bispecific antibody binding CD3 and CD133 by heteroconjugation. Using cytokine-induced killer cells, sufficient *in vitro* killing of CD133^+^ pancreatic (SW1990) and hepatic cancer (Hep3B) cancer cells was mediated with additional administration of the bispecific antibody. The concept of targeting CD133^+^ tumor cells and simultaneously binding CD3 was also shown to be efficient in an *in vivo* model after tumor inoculation (SW1190) and treatment [107]. Furthermore data revealed a relationship between treatment of CD133^+^ cancer cells with anti-CD3/anti-CD133 bispecific antibody-Cytokine Induced Killer (CIK) cells and S100P downregulation in the targeted tumor cells. S100P is a marker closely connected to tumor growth, migration and invasion [121,122], which indeed would have an additional and beneficial functionality in cancer defense. Taken together asymmetric bispecific antibodies, as well as, bispecific antibodies against CD133 and CD3 show good *in vivo* results and represent a promising direction in CSC treatment and elimination.

### 5.3. BiKES

#### CD16 × 133

As discussed, we believe NK activity and function can be improved by rendering them tumor specific. Recently, we developed a CD16 × 133 Bispecific NK-cell engagers (BiKEs) that simultaneously targets CD16 on NK-cells and CD133 on CSC [105]. This immune engager facilitates an efficient immunological synapse between the effector NK-cell and the tumor target of interest, triggering ADCC. This forced interaction of effector and target which results in activation of other NK-cell receptors like LFA-1/ICAM and thus promotes killing (Figure 1E). Efficacy was shown for CD133 expressing Caco-2 colorectal cancer cells in ^51^-Chromium release assays (Table 3). One drawback is that BiKES might be associated with adverse events due to sudden and extensive cytokine release after NK-cell activation [123]. Cytokine release was measured in this study. Only moderate production of IFN-γ and other toxic cytokines indicated that BiKEs might combine efficiency with a good safety profile [105,124].

### 5.4. Trispecific NK Engagers

#### 5.4.1. 133EpCAM16

**Figure 2:**
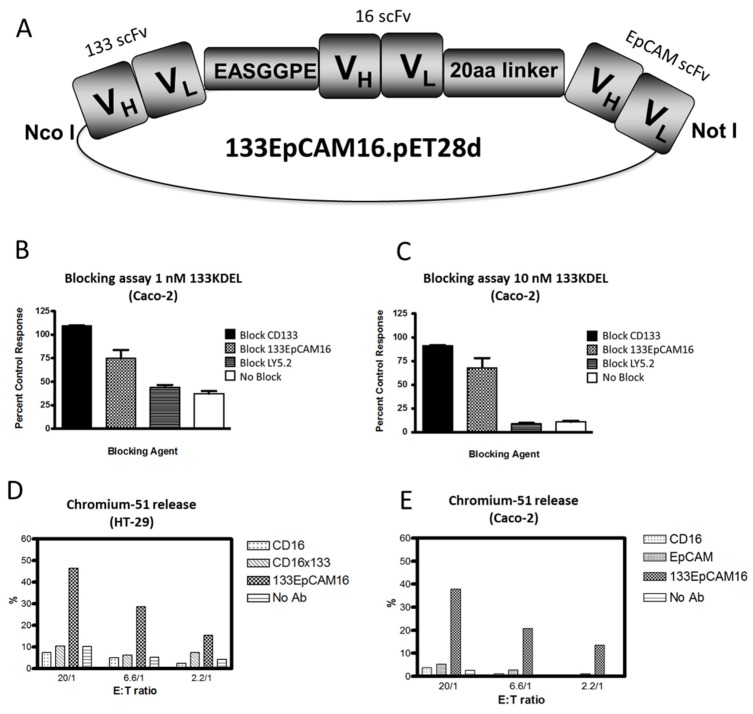
Engineering of 133EpCAM16 TriKE. (**A**) Construction of trispecific hybrid protein 133EpCAM16 NK-cell engager (TriKE). From left to right, the plasmid contains V_L_ and V_H_ regions of anti-133 scFv spliced to anti-CD16 scFv then to V_L_ and V_H_ regions of anti-EpCAM. The Figure shows all 3 of the ligands are active. To show that the anti-CD133 scFv was intact, we tested the ability of 133EpCAM16 to block the binding of the highly selective and potent targeted toxin CD133KDEL in (**B**,**C**). Caco-2 cells (40% CD133^+^, <95% EpCAM) were exposed to the targeted toxin 133KDEL in a concentration of 1 nM and 10 nM and blocked with 500 nM anti-CD133 scFv or 133EpCAM16 or negative control LY5.2 (anti-mouse CD45 antibody). The anti-CD133 scFv alone as well as the anti-CD133 scFv moiety of the TriKE sufficiently blocked CD133KDEL related killing in a tritiated thymidine uptake assay. To show that the anti-EpCAM scFv was intact, a ^51^Chromium release assay was performed with EpCAM^+^CD133^−^ HT-29 (**D**) and EpCAM^+^CD133^+^ Caco-2 (**E**) targets. 133EpCAM16 was able to kill HT-29, but a control CD16 × 133 scFv was not. Furthermore 133EpCAM16 was also able to kill Caco-2 targets whereas control scFvs did not. Since CD16 binding is required for the ADCC to occur, the anti-CD16 ligand was also intact.

Recently, we engineered a trispecific scFv, 133EpCAM16 (Figure 1F). This NK-cell engager binds CD16 on NK-cells, EpCAM, overexpressed on epithelial carcinomas, and CD133, expressed on CSC. The construct is illustrated in Figure 2A. The concept of 133EpCAM16 is analogous to the bispecific targeted toxin dEpCAMCD133KDEL. Two binding sites improve targeting of cancer cells which express both markers and also subpopulations in the same tumor bulk that express only one tumor related antigen such as mature carcinoma cells or CSC. To prove that the anti-CD133 scFv was intact, Caco-2 cells that express high levels of CD133 (40%–70% CD133^+^) (Table 4) were exposed to the targeted toxin CD133KDEL that comprises the same anti-CD133 scFv present in 133EpCAM16. Figure 2B,C show that 1nM or 10nM CD133KDEL inhibited proliferation of Caco-2 in a tritiated thymidine uptake assay after 72 h of drug exposure. The effect was mostly blocked with an anti-CD133 scFv or 133EpCAM16, but not with irrelevant control anti-LY5.2 antibody indicating that the binding capacity of the anti-CD133 scFv was intact and specific. To show the anti-EpCAM moiety of the TriKE was intact, EpCAM^+^ HT-29 cells that expressed only minimal levels of CD133 (Table 4) were incubated with effector cells in a chromium release assay that measures NK-cell killing (Figure 2D). 133EpCAM16 killed HT-29 targets but the highly active CD16 × 133 BiKE described in 5.2, did not, implying specificity of the EpCAM moiety (HT-29 only express a minimum of CD133). Thus, the killing observed on HT-29 was mostly attributed to the EpCAM moiety. Kd was not determined in these studies. ADCC could not occur without binding of the anti-CD16 moiety in this assay. Another chromium release assay was performed using Caco-2 cells. Whereas 133EpCAM16 induced killing, controls (an anti-EpCAM scFv, an anti-CD16 scFv) did not (Figure 2E). Together these data show that all three ligands selectively bound their intended targets.

#### 5.4.2. IL-15 TriKES

BiKEs have the ability to activate NK-cells and kill selectively via ADCC, but do not have the ability to expand the NK-cell population. In order to address this limitation, our research has focused on modified interleukins (IL) as a means to expand NK-cells participating in the anti-cancer response. IL-15 is known to have beneficial characteristics in cancer patients. Besides improving cytotoxicity and activation level, IL-15 can regulate and initiate anti-apoptotic and proliferative signals in NK-cells leading to enhanced expansion and survival [125,126,127,128,129]. IL-15 also has a lower level of unfavorable side effects such as capillary leak compared to IL-2, as shown in a murine model [130]. Recently, we published an IL-15 TriKE consisting of anti-CD16 scFv and anti-CD33 scFv [131]. A modified IL-15 cross-linker is flanked by both scFvs (Figure 1G). Our efforts focused on combining cytokine related NK-cell engaging effects with the binding capability to CD16 and CD33 resulting in a focused delivery of the IL-15 signal to the immunologic synapse for AML therapy. *In vitro* data showed superior anti-cancer activity and degranulation compared to 1633 without the IL-15 moiety [105] and a moderate cytokine release. *In vivo* experiments proved that the IL-15 TriKE and not the BiKE were capable of expanding NK-cells and inducing an anti-cancer response in a xenograft model. Since we have recently successfully developed a TriKE targeting EpCAM [129], we are currently synthesizing an IL-15 TriKE using the anti-CD133 scFv to target CSC. Combining NK-cell engagers with IL-15 might represent a new generation of drugs capable of self-sustaining NK effector cells leading to a new and effective immunotherapy of cancer.

### 5.5. Aptamers

In 1990, two independent study groups engineered new constructs based on nucleic acid construction capable of binding selected cell membrane targets [132,133]. Aptamers, as they are called, consist of short oligonucleotide bands of either RNA or DNA. Based on their biochemical nature, aptamers have some advantages over antibody constructs. Beside improved stability and a reduced immunogenic potential, synthesis is easier and less costly than protein engineering [134]. Further beneficial characteristics, such as improved tumor penetration because of their small size and effective epitope binding, led to engineering of constructs targeting typical epitopes such as CD33 on hematologic malignancies. These CD33 specific aptamers were sufficient in receptor related binding and also demonstrated internalization after binding in CD33^+^ myeloid leukemia lines making aptamers an attractive vehicle for specific targeted toxin delivery [135]. Thus, aptamers would have potential for targeting CSC. Shigdar *et al.* synthesized two RNA aptamers capable of binding the AC133 epitope and the CD133 protein. These aptamers showed superiority in tumor penetration, retention and internalization when compared to an AC133 specific antibody *in vitro* [136]. Taken together, aptamers show beneficial properties useful for efficiently targeting tumors.

## 6. Conclusions

Evidence grows that CSCs are present in an increased number of cancer entities comprising hematologic as well as solid cancer subgroups and are associated with relapses in patients after complete remission is achieved. This is mostly attributed to their chemotherapy and radiation resistance and implies the necessity of treatment options for this small but relevant cancer cell subpopulation.

Drug development focusing on elimination of CSC targeting CD133 is still at an early stage of development and clinical trials are limited. However, studies revealed that bystander killing of normal stem cells may not be a problem, implying that CD133 can be a promising target to eliminate CSC with low associated toxicity. Targeted toxins are a useful tool to eliminate CSC, however efficacy is dependent on the linked toxin and protease based enzymatic cleavage might also nonspecifically deliver toxins to healthy tissues. A new approach showed that nanoparticles can be effective in tumor treatment. Data about particle clearance and long term effects are not yet available indicating missing evidence of feasibility in a human setting. Our data showed that an alternative approach to killing CSC is to enlist the immune system using scFv constructs to harness NK-cells for elimination of CSC. To overcome the limitation of availability of NK-cell effectors, seen in BiKEs, implementation of IL-15 might further increase efficacy that has to be proved in clinical trials. Recent data imply that this development might fundamentally improve anti-cancer performance. By further improving this new self-sustaining mode of action, the new generation of NK-cell engagers has the potential to be a sufficient treatment modality on the way to kill CSC, the roots of cancer.

## Figures and Tables

**Table 1:** Cancer stem cell markers.

Marker	Source of Malignancy	Ref.
CD20	Melanoma	[39]
CD24	Nasopharyngeal	[40]
	Breast	[41]
	Pancreatic	[42]
CXCR4	Breast	[43,44]
	Glioma	[45]
	Lung	[46]
CD47	Bladder	[47]
	Breast	[48]
CD44	Bladder	[47]
	Colon	[49]
	Gastric	[50]
	Ovarian	[51]
	Pancreatic	[42]
CD117	Lung	[52]
	Ovarian	[53]
EpCAM	Colon	[54]
	Breast	[55]
HER2/ERBB2	Breast	[56]
	Ovarian	[57]
CD34	Acute myeloid leukemia	[1]

Abbrevations: CXCR4—C-X-C chemokine receptor type 4 negative, EpCAM—Epithelial cell adhesion molecule, HER2—human epidermal growth factor receptor 2.

**Table 2:** CD133 expression by different cancer subtypes.

Tissue Source	CD133^+^ Cell Group	Ref.	CD133^+^ Tumor Cells in Tumor Tissue
Breast	Cancer inducing subpopulation	[58,59]	Unknown
Colon	Cancer inducing subpopulation	[37,60]	2.5%
Prostate	Subpopulation	[38,61]	0.5%
Melanoma	Cancer inducing subpopulation	[62]	1%
Lung	Cancer inducing subpopulation	[63]	10%
HNSCC	Subpopulation	[64]	18%
Ovarian	Cancer inducing subpopulation	[65]	5.6%–16%
Pancreatic	Subpopulation	[66]/[67]	>1%/>15%
Gastric	Subpopulation	[68]	>1%
Hepathocellular	Subpopulation	[69]	1%–3%

Abbreviations: HNSCC—Head and Neck squamous cancer.

**Table 3:** CD133^+^ targeted cancer subtypes, used cell lines, results.

Drug	Cancer Subtype	Cell Line	Ref.	Result
^C178A^BC-CD133MAb	HNSCC	CAL-27	[64]	Inhibition of proliferation (*in vitro*)
dCD133KDEL	HNSCC	UMSCC-11B	[62]	Inhibition of proliferation, degradation (*in vitro*)
		NA-SCC		Inhibition of proliferation (*in vitro)*
	Ovarian	NIH:OVCAR5	[65]	Inhibition of growth (*in vivo*)
	Breast	MDA-MB-231	[102]	Inhibition of proliferation (*in vivo*)
DTEpCAMCD133KDEL	HNSCC	UMSCC-11B	[103]	Inhibition of proliferation and CR (*in vivo*)
	Colorectal	Caco-2		Inhibition of proliferation (*in vitro*)
		HT-29		Inhibition of proliferation (*in vitro*)
	Breast	BT-474		Inhibition of proliferation (*in vitro*)
		SK-BR3		Inhibition of proliferation (*in vitro*)
	Glioma	U87		No effect
	Lymphoma	Raji		No effect
CD133 NP	Colorectal	Caco-2	[104]	Particle uptake (*in vitro*)
	Breast	mammospheres		Cell elimination (*in vitro*)
		MDA-MB-231		Tumor decline (*in vivo*)
CD16 × 133	Colorectal	Caco-2	[105]	Cell elimination (*in vitro*)
	Lymphoma	Daudi		Cell elimination (*in vitro*)
MS133	Colorectal	HCT 116	[106]	Cell elimination (*in vitro*)/repression of tumor initiation (*in vivo*)
CD133CD3 bispecific antibody	Pancreatic	SW1990	[107]	Cell elimination/inhibition of tumor growth (*in vivo*)
	Hepatic	Hep3B		Cell elimination

Abbrevations: Ref. reference, HNSCC Head and Neck cancer, CR complete remission, NP nano particle.

**Table 4:** Flow cytometry based evaluation of target cell expression.

Cell Line	Dose (µg)	Anti-CD133-FITC (%)	Anti-EpCAM-FITC (%)	Anti-CD16-FITC (%)
Caco-2	0.5	40.0	99.6	-
	1	41.5	99.7	-
	2	48.0	99.7	0.6
HT-29	1	0.8	98.2	-
	2	1.4	99.2	0.8

Abbrevations: -: negative, FITC—fluorescein isothiocyanate.
